# Challenges in the terminology of second-degree atrioventricular block

**DOI:** 10.1007/s00399-024-01055-5

**Published:** 2024-11-18

**Authors:** S. Serge Barold, Carsten W. Israel, Bengt Herweg

**Affiliations:** 1 Clinical Professor of Medicine, University of Rochester School of Medicine, University of Rochester School of Medicine and Dentistry, Rochester, NY USA; 2 Campus Bielefeld-Bethel, Evangelisches Klinikum Bethel gGmbH, Universitätsklinikum OWL der Universität Bielefeld, Bielefeld, Germany; 3 Professor of Medicine, University of South Florida, 33606 Tampa, FL USA

**Keywords:** Mobitz type II atriventricular block, Heart block, Electrocardiography, Arrythmia, Mobits type I atrioventricular block, AV-Block II°, Typ Wenckebach, AV-Block II°, Mobitz Typ II, Atrioventrikulärer Block zweiten Grades, AV-Block II°, Mobitz Typ I, Atrioventrikulärer Block

## Abstract

The terminology of second-degree atrioventricular block has evolved over time, thereby creating some confusion and misinterpretations. Strict adherence to standard terminology and the appropriate use of eponyms are important to avoid diagnostic errors.

## Abstract (de)

Die Terminologie des atrioventrikulären Blocks zweiten Grades hat sich mit der Zeit weiterentwickelt, wodurch einige Verwirrung und Fehlinterpretationen entstanden. Die strikte Beachtung der Standardterminologie und der korrekte Gebrauch der Namensgeber sind wichtig, um diagnostische Fehler zu vermeiden.

In 1873, using an isolated frog heart preparation, the Italian physiologist Luigi Luciani was the first to demonstrate group beating [1]. In 1899, Wenckebach credited Luciani with this discovery and called the phenomenon “Luciani periods” [2]. This form of group beating has become known as Wenckebach periodicity in second-degree atrioventricular block (AVB). Thus, Wenckebach AVB is sometimes called Luciani–Wenckebach periodicity/AVB.

“Wenckebach” is one of the most common eponyms in medicine. The form of AVB described by Wenckebach is now called traditional or classic. The mathematical characteristics of the classic form are well-known. The atypical type has also been called “common” since it is far more frequent than the traditional form. Atypical Wenckebach AVB has never been clearly defined by professional organizations. It can be considered a form of Wenckebach AVB that deviates in any way from the traditional form. Wenckebach AVB is often labeled as Wenckebach type I AVB, but it is an example of tautology, given that there is no need to add “type I” since the term “Wenckebach AVB” is self-evident. Furthermore, using the label of type I incorrectly suggests to the beginner that in Wenckebach AVB, there might also be a type II Wenckebach AVB.

In 1924, using the electrocardiograph, Mobitz classified the well-known Wenckebach form of second-degree AVB as type I and characterized the form of AV block originally described by Hay in 1906 as type II second-degree AVB [3, 4]. This led to an alternative appellation of Wenckebach AVB as Mobitz type I AVB. Unfortunately, use of the term “Mobitz block” in the literature, presumably referring to type II block, is potentially misleading without specifying whether it refers to Mobitz type I or type II AVB. Mobitz did not specifically indicate that sustained 2:1 AVB was a form of type II AVB. Rather, he showed that 2:1 AVB may be associated with sequences showing intermittent typical type II AVB. Unfortunately, to this day, sustained 2:1 AVB is not infrequently called Mobitz type II AVB in the literature. Furthermore, high grade or advanced second-degree AVB with constant PR intervals before and after the non-conducted P waves is called Mobitz II AVB by purists, who cite Mobitz’s original article showing more than one blocked P wave, thereby defying the present codified definition requiring the block of a single P wave. In other words, apart from Mobitz type I and II AVB, any other form of second-degree narrow QRS AVB should not be automatically called Mobitz type I block, while any form of wide QRS complex second-degree AVB should not automatically be labeled as Mobitz type II block.

The term “apparent Mobitz AVB” has been used to describe some cases of vagally induced AVB associated with sinus slowing. “Pseudo-Mobitz type II AVB” has been used to describe a manifestation of concealed extrasystoles mimicking true Mobitz type II AVB [5]. It has also been applied to a sequence of Wenckebach AVB with constant PR intervals in its terminal part before the non-conducted beat mimicking true Mobitz type II AVB. The term “pseudo-Mobitz type II AVB” seems preferable to “apparent.”

## Dual occurrence of eponyms

Terminology becomes complicated when true Mobitz type II AVB is associated with pseudo-Mobitz type II AVB in the same patient. This may occur in patients with His-Purkinje disease causing both true type II and pseudo-type II [6]. The latter is caused by associated concealed ventricular extrasystoles known to occur with this kind of pathology. In contrast, both Mobitz type I (Wenckebach) and true Mobitz type II AVB rarely occur together in sequential electrocardiograms. Only two such cases have been reported [7, 8]. Both occurred in women with a narrow QRS complex. In both cases, electrophysiologic studies revealed split His bundle potentials. This situation is unrelated to the rare phenomenon of alternating Wenckebach periodicity when the two levels of block are in the atrioventricular node and the His-Purkinje system [9].

## Combined Mobitz and Wenckebach appellation

Wenckebach AVB is often called Mobitz type I AVB. However, when teaching or giving oral presentations using only the terms of Mobitz type I and Mobitz type II AVB, their distinction may sometimes become blurred for the audience. If an individual insists on using only the Mobitz eponym for the two types of AVB, it might be helpful to say “Mobitz type I.-Wenckebach” AVB to prevent confusion. Obviously, this strange combination should never be used in a written discourse.

## Stand-alone type I and type II AVB

There is no such thing as stand-alone “type I” and “type II” AVB. However, if these terms are to be used in a publication, it should be clearly stated that they refer to Wenckebach (Mobitz type I) or Mobitz type II second degree AVB.

Finally, the Europeans often describe second degree AVB as II°AV block. At first glance, it may appear that AV block II° represents Mobitz type II AVB.

## Conclusion

Second-degree AVB is the most common erroneous arrhythmia diagnosis made by medical students, residents-in-training, and even physicians. Strict adherence to terminology and the use of appropriate eponyms are important to avoid diagnostic errors.

## Declarations

For this article no studies with human participants or animals were performed by any of the authors. All studies mentioned were in accordance with the ethical standards indicated in each case.
